# Adhesins in the virulence of opportunistic fungal pathogens of human

**DOI:** 10.1080/21501203.2021.1934176

**Published:** 2021-07-05

**Authors:** Amrita Kumari, Ankita H. Tripathi, Poonam Gautam, Rekha Gahtori, Amit Pande, Yogendra Singh, Taruna Madan, Santosh K. Upadhyay

**Affiliations:** Department of Biotechnology, Sir J.C. Bose Technical campus, Kumaun University, Nainital, India; ICMR-National Institute of Pathology, New Delhi, India; Directorate of Coldwater Fisheries Research (DCFR), Nainital, India; Department of Zoology, University of Delhi, New Delhi, India; ICMR-National Institute for Research in Reproductive Health (NIRRH), Mumbai, India

**Keywords:** Adhesin, adherence phenomenon, adhesion, host–pathogen interaction, morphotypes, GPI-anchored proteins, infectious propagules

## Abstract

Aspergillosis, candidiasis, and cryptococcosis are the most common cause of mycoses-related disease and death among immune-compromised patients. Adhesins are cell-surface exposed proteins or glycoproteins of pathogens that bind to the extracellular matrix (ECM) constituents or mucosal epithelial surfaces of the host cells. The forces of interaction between fungal adhesins and host tissues are accompanied by ligand binding, hydrophobic interactions and protein-protein aggregation. Adherence is the primary and critical step involved in the pathogenesis; however, there is limited information on fungal adhesins compared to that on the bacterial adhesins. Except a few studies based on screening of proteome for adhesin identification, majority are based on characterization of individual adhesins. Recently, based on their characteristic signatures, many putative novel fungal adhesins have been predicted using bioinformatics algorithms. Some of these novel adhesin candidates have been validated by *in-vitro* studies; though, most of them are yet to be characterised experimentally. Morphotype specific adhesin expression as well as tissue tropism are the crucial determinants for a successful adhesion process. This review presents a comprehensive overview of various studies on fungal adhesins and discusses the targetability of the adhesins and adherence phenomenon, for combating the fungal infection in a preventive or therapeutic mode.

## 1. Fungal adhesins

Adhesion of pathogenic microbes to host tissue is the primary step of infection and is crucial for the persistence of the pathogen in the host (Pwj et al. 2013). This phenomenon is mediated by microbial cell-surface molecules called adhesins that promote attachment of microbes to the host tissue. Specificity of infecting (more efficiently) a particular host or tissue (tissue tropism) is determined by interactions between adhesins and their specific receptors on surface of the host-tissue (Klemm and Schembri 2000). Fungal adhesins are mostly proteins or glycoproteins, and both the carbohydrate or protein parts of these molecules have been entailed in interacting with the host-tissue/-molecules to facilitate adhesion (Tronchin et al. 2008). Role of fungal adhesins in infecting and colonising host has been established by various researchers. The most notable of these studies involves constructing deletion mutants of putative adhesins, or blocking of their function to demonstrate the resultant decline in the adherence and virulence of fungal morphotype(s). In mycology, the most widely studied adhesins are of *Candida albicans*; however, in recent times new adhesins have been identified for most of recurrently occurring fungal infections, caused by the other *Candida spp., Aspergillus fumigatus (Afu), Blastomyces dermatitidis, Coccidioides immitis, Cryptococcus neoformans, Paracoccidioides brasiliensis,* and *Histoplasma capsulatum* (Perlroth et al. 2007).

Cell wall components of fungi provide rigidity and protect them against environmental constrains (Paul 2007). Carbohydrates like chitin, β-1,3/1,6-glucan comprise approximately 60−70% of the total mass of cell wall in *Candida spp*. Proteins in the cell wall fungi are mostly linked covalently to β-glucan through GPI anchor. Majority of these cell-wall proteins are implicated in the regulation of primary host–pathogen interactions, as adhesins, aspartyl proteases, dismutases, and phospholipases (Pwj et al. 2013). Biotechnological advancements and use of sophisticated techniques have enhanced our understanding regarding the role of fungal adhesins in mediating adhesion, aggregation, biofilm formation, and inhibiting the fungus specific host immune response. Most of characterised adhesins are proteins with a relatively complex N-terminal domain that mediates specific peptide–peptide, peptide–sugar and other peptide–ligand interactions (Klis et al. 2001). The GPI-anchored adhesins comprise of conserved sequences: an N-terminus endoplasmic reticulum (ER) signal peptide and a C-terminus peptide for GPI-ylation in the ER membrane (Verstrepen and Klis 2006). Downstream the N-terminal domain resides a variable domain with low complexity and abundance of serine/threonine tandem repeats. Greater adherence capacity of these glycoproteins is attributed to presence of longer repeat regions, whereas shorter repeats culminate in decreased adherence, possibly because the effector N-terminal domain remains engraved in the cell wall in such cases (Verstrepen and Klis 2006).

## 2. Opportunistic fungal pathogens and invasive infections

Mycoses infecting immunocompromised individuals but not the healthy ones come under the category of opportunistic human fungal pathogens. They can cause a wide range of complications including mild to severe, localised to systemic and superficial to invasive infections. Invasive fungal infections (IFIs) can cause severe deep tissue, disseminated bloodstream and respiratory tract infections (Warnock 2007). According to revised definition of IFIs proposed by “*EORTC/MSG Consensus Groups*”, they are categorised into three groups: 'proven', 'probable', and 'possible', based on certain defined diagnostic criteria (B J et al. 2018). Pathogenic fungi are leading cause of many superficial, mucosal and invasive infections among humans. In the iatrogenic environment, there is a high incidence of IFIs, particularly in the people with compromised immune system. IFIs are arduous to diagnose and treat, and therefore, lead to higher rates of morbidity and mortality. Marked risk of infection is associated with the immunocompromised individuals with neutropenia, solid organ or bone marrow transplants, HIV infection, haematological disorders and patients undergoing chemotherapy and immunosuppressant drug therapies (Aikawa et al. 2005; Baddley 2011; Badiee and Hashemizadeh 2014). Approximately, 90% of the documented IFI-related mortality is caused by ten notorious species of fungi including: *Aspergillus fumigatus* (Aspergillosis), *Blastomyces dermatitidis* (Blastomycosis), *Candida albicans* (Candidiasis), *Cryptococcus neoformans* (Cryptococcosis), *Rhizopus oryzae* (Mucormycosis), *Coccidioides immitis* (Coccidioidomycosis), *Pneumocystis jirovecii* (Pneumocystis), *Histoplasma capsulatum* (Histoplasmosis), *Paracoccidioides brasiliensis* (Paracoccidioidomycosis), *Penicillium marneffei* (Penicilliosis) (Brown et al. 2012).

As a causative agent of invasive infection, the fungus must first adhere to the mucosal tracts and then, actively invade the tissue with the help of enzymatic and cytopathic virulent factors. Morphological transformation strategies are adopted by many fungal pathogens in the course of ensuing invasive infection. Several humans opportunistic mycoses are dimorphic and capable of undergoing morphogenetic transition between their yeast and hyphal forms, thereby inclining them as per the requirement of phases of colonisation in host and invasion. Four genera of fungi, *Candida, Aspergillus, Cryptococcus* and *Pneumocystis*, are known to be responsible for approximately 90% of all fungal-infection-related deaths (Brown et al. 2012; Shourian and Qureshi 2019).

*Candida albicans* is known to display prominent adherence ability to the host-tissue. About 25–30% of cancer/leukaemia patients enduring chemotherapy are predisposed to oropharyngeal candidiasis (Rolston and Bodey 2003). Dissemination of fungi in the blood stream of leukaemic patients with oropharyngeal candidiasis can lead to its systemic form, which makes it more cumbersome to treat(Degregorio et al. 1982). *C. albicans* strains have been reported to adhere, colonise and disseminate the murine alimentary tract devoid of intestinal bacteria (Westwater et al. 2007). Different morphotypes of *C. albicans* including spore, hyphae and pseudo-hyphae interact differently with host immune cells. Switching of morphotypes of *C. albicans* between the spore and hyphal forms is associated with its virulence. Cell wall structures of the hyphal form(s) are distinct from the spore-form. The hyphae of *C. albicans* cause profound damage to the host tissues, whereas the spores are efficient in evading the host immunity. Among total incidences of acute and disseminated candidiasis most of the infections are manifested by four species of *Candida*, namely, *C. albicans, C. glabrata, C. parapsilosis* and *C. tropicalis* (Hajjeh et al. 2004; Pfaller and Diekema 2004a). *C. glabrata* has been documented as a rapidly emerging non-albicans species of *Candida* resistant to azole antifungal agents (Pfaller and Diekema 2004a), whereas, relatively smaller fraction of *Candida*-related blood stream infections are known to be driven by *C. guilliermondii, C. krusei, C. dubliniensis, C. lusitaniae and C. rugosa* (Pfaller and Diekema 2004a).

*Aspergillus fumigatus*, a mould belonging to the eurotiomycetes class, is the most frequent opportunistic human pathogen among other genera of the class and is known to cause allergic bronchopulmonary aspergillosis (ABPA), aspergilloma, and acute invasive aspergillosis (IA) infections in immunocompromised individuals with high rates of morbidity and mortality (Latgé 2003; Beauvais et al. 2007). The pathogen is often characterised by greenish cone-shaped conidia, from 2.5 to 3 µm in diameter, produced in chains, whereas some isolates of *A. fumigatus* have no pigments and produce white conidia (J P Latgé 1999). *A. fumigatus* is identified as the most abundant fungal pathogen in the air, causing serious and generally fatal invasive infections in immunocompromised individuals (Andriole 1993), wherein the spores (conidia) of *A. fumigatus* may establish an invasive disease in the lung tissue by the development of hyphae. The hyphae grow by the extension of the tips that generates significant pressure on the tip, enough for penetrating the substrata (Money 2001). Exact data have not been recorded; however, it has been predicted that fungal hyphae are capable of exerting a total force equivalent to 0.01–0.1 N/m^2^ on their hyphal tips. For comparison, 0.1 N/m^2^ is the opposite resistance force exerted by an 8% agar (w/v) gel (Hube and Naglik 2001). Additionally, the tips of hyphae also secrete hydrolysing enzymes capable of degrading host-cellular and molecular components that facilitate the infiltration of growing hyphae into the surrounding host-tissue (Gow et al. 2002). Hope *et al*. developed a double-layer model of the pulmonary alveolus with lung endothelial cells cultured at the bottom of the cell culture inserts and a type II pneumocyte line cultured on the upper plane. On adding *A. fumigatus* conidia to the upper chamber of this model, the conidia germinated and formed hyphae that subsequently invaded the abluminal surface of endothelial cells (Hope et al. 2007). The invasion of *A. fumigatus* from the luminal surface of the endothelial cells is mediated by the formation of pseudopodia that enveloped the hyphae. The formation of these pseudopodia is accompanied by the accumulation of a dense network of microfilaments around the endocytes (Kamai et al. 2009). Other common *Aspergillus* species responsible for causing allergic and invasive aspergillosis in humans include *A. flavus. A. niger* and *A. terreus* (Krishnan et al. 2009).

*Cryptococcus neoformans* is ubiquitous opportunistic aetiological agent found in soil dwellings, avian excreta and air, causing pulmonary cryptococcosis in immunocompromised individuals upon exposure of its spores. The most commonly manifested cryptococcal infection is cryptococcal meningoencephalitis of brain that is associated with approximately 50% mortality (Williamson 2017). The hallmark feature of *C. neoformans* is a polysaccharide capsule that surrounds the cell body. In immuno-compromised individuals (HIV patients, recipients of bone marrow and solid organ transplants) exposure of cryptococcal spores from environment causes the fungus to colonise the host’s alveolar tissue causing characteristic inflammation and nodulation in the lungs. After evading the exerted host defence, it can gradually disseminate to other organs including the central nervous system through breaching blood brain barrier and causing inflammation in meninges layer. The initial infection is latent without any notable symptoms resulting in more severe conditions in later stages of disseminated systemic infection. Besides *Cryptococcus sp*., the earlier mentioned invasive mycoses can also cause brain infections like meningitis. The outer capsular components protect *C. neoformans* from phagocytosis by alveolar macrophages and support their persistance inside, coping with oxidative stress and breach the blood brain barrier through transcytosis by brain microvascular endothelial cells (BMECs) (Shourian and Qureshi^2019^; Brown et al. 2012; Liu et al. 2012).

*Pneumocystis jirovecii* (aka, *P. carinii)* was discovered in HIV patients in 1980. Initially, it was misidentified as a protozoan but is now recognised as a fungus (Edman et al. 1988). *Pneumocystis carinii* pneumonia (PCP) is one of the major opportunistic mycotic infections of AIDS manifested patients and is considered most common AIDS defining illness of the modern era (Fei et al. 2009). Interestingly, the mortality of PCP is 10% in HIV-positive patients and 30–60% in HIV-negative immunocompromised patients. An abnormally intense inflammatory response in HIV-negative patients is believed to be responsible for the higher mortality in these cases (Mansharamani et al. 2003; Kotani et al. 2017).

Zygomycetes infections are rare, reaching an annual rate of 1.7 infections per million people, inside the United States (Rees et al. 1998 Pfaller and Diekema 2004a). Infections with these organisms are typically acute and unexpectedly progressive, with mortality rates of 70–100% (Gonzalez et al. 2002). The most ordinary etiologic mediator of zygomycosis encompasses members of the genera *Rhizopus, Mucor* and *Absidia*. These organisms lead to infections in immunocompromised people, particularly with ketoacidosis, diabetes neutropenia and corticosteroid therapy (Gonzalez et al. 2002). Zygomycetes have a marked tendency for blood vessels (angioinvasion) and produces numerous emboli with associated myocardial infarction and contiguous tissue necrosis (Pfaller and Diekema 2004b).

*Penicillium marneffei*, a dimorphic fungus, causes penicilliosis in healthy as well as immunocompromised individuals and is mostly endemic to Southeast Asian countries. This fungus was first identified in bamboo rats for manifesting infection. Later on, it was found to infect HIV infected individuals in endemic regions causing cutaneous skin lesions. The mortality rates are high because of poor early diagnosis and prophylaxis (Sirisanthana and Supparatpinyo 1998).

*Histoplasma capsulatum* is the causative agent of acute pulmonary histoplasmosis and disseminated Histoplasmosis. Repeated exposure to the spores by inhalation results in acute pulmonary histoplasmosis affecting host’s respiratory tract. When the immune cells of host are unable to clear the pathogen or the fungus adopts certain escape tricks to host immune clearance mechanisms, it disseminates systemically, leading to more severe disseminated Histoplasmosis (Goodwin et al. 1980).

Blastomycosis is another opportunistic infection caused by dimorphic fungus *Blastomyces dermatitidis*. This infection is mostly prevalent in the riverside regions of Mississippi and Ohio rivers in America and Canada (Khuu et al. 2014). Common habitats for this fungus include soil with dead and decaying materials. Spores are predominantly air-borne and come in contact with host respiratory system by inhalation. When host defence mechanisms are insufficient to combat, it undergoes morphogenetic transition to colonise the infected tissue and further disseminates to other organs, leading more severe systemic spread (Saccente and Woods 2010).

Besides these common species of popular opportunistic fungi, in the recent years, several less virulent strains of *Aspergillus, Candida, Cryptococcus* and *Histoplasma* have attained higher virulence. Some of emerging fungi leading to recent outbreaks includes *Candida auris*, previously known for causing superficial fungal infection, such as otitis, is now adapted to cause more severe invasive diseases like bloodstream infections (BSI). *C. auris* mediated bloodstream fungemia has been recorded in many countries mainly in South Korea (Lee et al. 2011), India (Anuradha Chowdhary et al. 2013), South Africa (Magobo et al. 2014), Kuwait (Emara et al. 2015), Pakistan (A. Chowdhary et al. 2014) and Venezuela (Calvo et al. 2016). This strain is resistant to the first line antifungal agents viz., fluconazole, amphotericin B and echinocandins (Lockhart et al. 2017). Another fungus with notable emergence of virulence is *Cryptococcus gattii* with its two serotypes (B and C) (KJ and Varma 2006) and four molecular types (VGI, VGII, VGIII and VGIV). Whole genome sequencing of this strain suggested its close identity with C*ryptococcal neoformans*. However, unlike *C. neoformans*, which is known to target immune suppressed individuals, *C. gattii* has predilection for individuals with intact immunity (Shourian and Qureshi 2019). *C. gattii* outbreaks have already been reported from North American Pacific Northwest region (Perfect 1989), tropical northern Australia (Fisher et al. 1993), Papua New Guinea (Seaton 1996), sub Saharan Africa (Litvintseva et al. 2005), Vancouver Islandand Western Canada (Kidd et al. 2004); however, its occurrence is believed to be more widespread (Byrnes et al. 2011). Different studies suggested an overall mortality rate of 13–33% among patients infected with *C. gatti* (https://www.cdc.gov/fungal/diseases/cryptococcosis-gattii/statistics.html).

Looking at the statistics for the prevalence of these fungal infections suggests that each year >200,000 immunocompromised individuals are affected with invasive aspergillosis with more than 50% (in high risk patients) estimated mortality (Garcia‐Vidal et al. 2008; Guinea et al. 2010). Approximately, 3 million individuals worldwide are affected with chronic pulmonary aspergillosis, especially patients with preexisting health conditions, such as, asthma and other pulmonary diseases, where it results in nearly 15% mortality (Nam et al. 2010; Denning et al. 2011). More than 52,000 deaths per annum have been recorded in patients suffering from *Pneumocystis jirovecii* pneumonia/Pneumocystis, especially in HIV patients (Walzer et al., 2008). A fatal mycotic brain infection is caused by *Cryptococcus meningitis* and as estimated by U.S. Centre for Disease Control and Prevention (CDC), every year approximately 220,000 new cases of cryptococcal meningitis are recorded with nearly 181,000 deaths of patients. The situation worsens in some developing countries with poor medical facilities and higher prevalence of immunocompromised individuals particularly due to HIV infection. Most of the deaths because of cryptococcal meningitis in the recent past were recorded in sub-Saharan Africa (https://www.cdc.gov/fungal/diseases/cryptococcosis-neoformans/statistics.html). Worldwide, disseminated *Candida* blood stream infections are diagnosed in approximately 400,000 individuals each year. Estimated and recorded mortality rates for these infections are 42 % and 27% respectively (Morgan 2005; Brown et al. 2012).

Coccidioidomycosis, also called “valley fever”, is caused by the exposures to spores of soil dwelling fungus *Coccidioides immititis*. This fungus is endemic to arid regions of Mexico and United States. Approximately, 111,717 cases of coccidioidomycosis were reported during the years 1998–2011 from different states of US (“Increase in Reported Coccidioidomycosis – United States, 1998–2011,” 2013).

**Figure 1.**
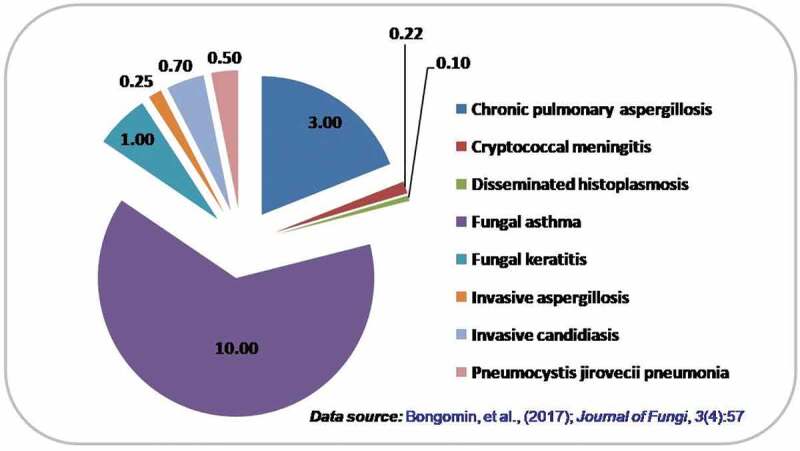
Estimated global burden of fungal diseases annually (in millions)

A summary of estimated global burden of invasive opportunistic fungal diseases is displayed in Figure 1.

## 3. Characterised adhesins of common opportunistic human fungal pathogens

*C. albicans, A. fumigatus, Sporothrix schenckii, C. glabrata, C. neoformans* and *Paracoccidioides brasiliensis* are among the few opportunistic fungal pathogens in which the adhesion phenomenon is relatively better studied and certain adhesins have been characterised. For many other common opportunistic fungi, adhesins have sparingly been studied. Table 1 is a compilation of adhesins identified in common opportunistic human fungal pathogens.

**Table 1.** List of characterised adhesins from opportunistic fungal pathogens

Fungus	Adhesin	Morphotype	Ligand	References
*Aspergillus fumigatus*	RodA	Conidia	Collagen	Thau et al. (1994)
	37 and 72-kDa proteins	Conidia	Laminin	Tronchin et al. (1997)
	32-kDa protein	Conidia	Fucose	Mendes-Giannini et al. (2000)
	Mp1 (Galactomannoprotein)	Swollen conidia	ECM	Upadhyay et al. (2008)
	Extracellular Thaumatin domain protein (AfCalAp)	Swollen conidia	Laminin, Fibrinogen, Mice Lung cells	Upadhyay et al. (2009); Liu et al.(2016)
	Hydrophobins	Conidia	ECM	Abad et al. (2010)
	CspA	Conidia	ECM	Levdansky et al. (2010)
	Alpha-mannosidase	Swollen conidia	Fibrinogen	Upadhyay et al.(2012)
	Amidase familyprotein, putative			
	Beta-glucosidase, putative			
	Hypothetical protein			
	Pectate lyase A			
	Oryzin precursor (Alkaline proteinase) (Elastase)			
*A. niger*	HF-extractable cell wall putative GPI-anchored mannoprotein – cwpA	Conidia	ECM	Damveld et al. (2005)
Blastomyces dermatitidis	120-kDa protein (WI1)/BAD1g	Yeast form	ECM proteins	Brandhorst et al. (1999)
*Candida albicans*	55- and 105-kDa proteins	Yeast form	Fibronectin (RGD domain)	Sa et al. (1992)
	37-kDa	Yeast form	Laminin	Lo´pez-Ribot et al. (1994)
	60- and 105-kDa glycoproteins	Yeast form	Fibronectin	Klotz et al.(1994)
	68-, 62- and 60-kDa proteins	Yeast form	Laminin	Lo´pez-Ribot et al. (1994)
	Glyceraldehyde-3-phosphate dehydrogenase (GAPDH)	Yeast form	fibronectin and laminin	Gil-Navarro et al. (1997); Gozalbo et al. (1998)
	Als1p, Ala1p/Als5p	Yeast form	ECM proteins	Sundstrom (1999)
	Hwp1	Yeast form	Mammalian transglutaminases	Staab et al. (1999)
	EAP1g	Yeast form	Epithelial cells	Li and Palecek (2003)
	Int1	Yeast form	ECM proteins	de Groot et al. 2004; Zhao et al. 2004
	ALS (Als 1p-Als9p),	Yeast form	ECM proteins	de Groot et al. 2004; Zhao et al. 2004
	EPA and GPI dependent CWPs	Yeast form	ECM proteins	de Groot et al. 2004; Zhao et al. 2004
	CaIff4	Yeast form	Epithelial cells; Plastic surface	Kempf et al. 2007
	Alcohol dehydrogenase (Adh1)	hyphae	Vitronectin, fibronectin, laminin	Kozik et al. 2015
	Phosphoglycerate mutase (Gpm1)	hyphae	Vitronectin	Kozik et al. 2015
*Candida glabrata*	Epa1p/EPA1g	Yeast form	Host-cell carbohydrates	Cormack et al. 1999
*Candida tropicalis*	Surface expressed integrin analogue (putative fibronectin receptor)	Yeast form	Fibronectin	Cm and Hostetter 1993
	105-kDa surface expressed fibronectin-binding protein	Yeast form	ECM proteins	Gp and Hostetter 1996
	Fibronectin-binding proteins gi|255722,852 gi|255,728,333 gi|255,727,881 gi|255,732,698 gi|255,722,021	Pseudohyphae	fibrinogen	Kozik et al. 2015
	Vitronectin-binding proteins gi|255,722,852 gi|240,134,900 gi|255,728,333 gi|255,727,881 gi|255,732,698 gi|255,722,021	Pseudohyphae	Vitronectin	Kozik et al. 2015
	Laminin-binding proteins gi|255,722,852 gi|240,134,900 gi|255,728,333 gi|240,132,975 gi|255,727,881 gi|255,732,698 gi|255,722,021	Pseudohyphae	Laminin	Kozik et al. 2015
*Candida parapsilosis*	Surface Als like proteins	Pseudohyphae	ECM proteins	Kozik et al. 2015
	Fibronectin-binding proteins gi|354,547,941, gi|354,547,939 gi|354,547,813 gi|354,545,198 gi|354,545,113 gi|354,544,475 gi|354,546,116 gi|354,545,980 gi|354,546,805	Pseudohyphae	Fibronectin	Kozik et al. 2015
	Vitronectin-binding protein gi|8,927,048 gi|354,545,198 gi|354,545,113 gi|354,544,475 gi|354,546,116 gi|354,545,980 gi|354,546,348 gi|354,545,888	Pseudohyphae	Vitronectin	Kozik et al. 2015
	Laminin-binding proteins gi|354,547,939 gi|354,545,113 gi|354,544,475 gi|354,546,805	Pseudohyphae	Laminin	Kozik et al. 2015
*Cryptococcus neoformans*	MP 15, 98, 88 and 84 kDa proteins	Yeast cells	Lung epithelial cells	Teixeira et al. 2014
	Cfl1	Hyphae	Plastic surface	Wang et al. 2012
*Coccidioides immitis*	SOWgpg/rSOWp	Parasitic phase	ECM proteins	Hung et al. 2002
*Fusarium oxysporum*	β-1,6 glycosyated GPI-CWP -Fem1p (60-kDa)	Microconidia	ECM	Schoffelmeer et al. 2001
*Fonsecaea pedrosoi*	50-kDa protein	Conidia	Manose and N-acetilglycosamine	Limongi et al. 2001
*Histoplasma capsulatum*	50-kDa protein	Yeast cells	Laminin	McMahon et al. 1995
	Heat shock protein (Hsp60)	Yeast cells	CD18 receptors on Macrophage cells, CHO cells	Long et al. 2003
*Metarhizium anisopliae*	MAD1 and MAD2	Conidia	ECM	Wang et al. 2007
*Penicillium marneffei*	20-kDa protein	conidia	Laminin and Fibronectin	Hamilton et al. 1998
	Cell Wall Mannoprotein Mp1p (58-kDa)	Yeast cells	Concanavalin A (a type of lectin purified from jack beans, binds with mannse residues of glycoproteins)	Cao et al. 1998
	Glyceraldehyde-3-phosphate dehydrogenase (GAPDH)	Conidia	A549 pneumocytes, fibronectin and laminin	Lau et al. 2013
*Phytophthora cinnamomi*	220 kDa-proteins with thrombospondin type 1 repeats	Zoospores	ECM	Robold and Hardham 2005
*Paracoccidioides brasiliensis*	30-kDa; 19- and 32-kDa proteins	Yeast cells	Laminin and ECM proteins	Andreotti et al. 2005
	Glyceraldehyde-3-phosphate dehydrogenase (GAPDH)	Yeast cells	fibronectin, laminin, and type I collagen	Barbosa et al. 2006
	43-kDa protein	Yeast cells	Laminin	Vicentini et al. 1994
	Pb 14-3-3 protein	Yeast phase	*S. cerevisiae* cells expressing Pb 14-3-3 adhere to epithelial cell-line A549	de Oliveira et al. 2015
*Pseudallescheria boydii*	Peptidorhamnomannan (PRM)	Conidia	HEp2 cells (human larynx carcinoma cells)	Pinto et al. 2004
*Pneumocystis jirovecii*	Msg protein	Yeast cells	A549 cell-line	Kutty et al. 2013
*Rhizopus oryzae*	Conidial surface adhesins	Spores and germ tube	HUVECs, ECM proteins, endothelial cells	Ibrahim et al. 2005
*Sporothrix schenckii*	Cell wall proteins with MW 90 and 135 kDa	Yeast cells	Human umbilical vein endothelial cells (HUVECs)	Figueiredo et al. 2004
	Gp70, Unidentified proteins in 37–92 kDa range	Yeast cells	Fibronectin	Teixeira et al. 2009
	Gp70 glycoprotein	conidia	Dermis of mouse tail	Ruiz-Baca et al. 2009
	Cell surface glycoconjugates with carbohydrate or peptide moieties	Yeast and conidia	Fibronectin	Lima et al. 2001
	Cell wall proteins with MW ≥ 190, 180, 115, 90 and 80 kDa	Yeast form	Epithelial cells	Sandoval-Bernal et al. 2011
*Trichophyton mentagrophytes*	Carbohydrate specific adhesins on microconidial surface	Microconidia	Mannose residues on Chinese hamster ovary (CHO) cells	Esquenazi et al. 2003
*Trichophyton rubrum*	Lectin-like adhesins on microconidial surface	Microconidia	CHO cells, epithelial and macrophages cells.	D. Esquenazi et al. 2004

### 3.1 Aspergillus fumigatus

**Figure 2.**
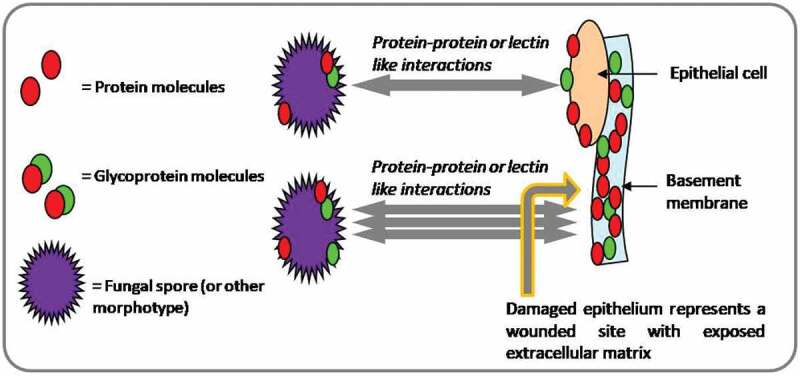
Extracellular matrix (ECM) accessible at the damaged epithelial sites facilitate more pronounced adhesion of fungal morphotypes to host-tissue(s)

*Spores of A. fumigatus* are capable of adhering to substrates of various nature, including host-cells, receptors and extracellular matrix (ECM) proteins, through polysaccharides, glycoproteins and proteins that cover their cell wall (Figure 2) (Bouchara et al. 1999; Wasylnka and Moore 2000). This filamentous opportunistic fungus also secretes a variety of proteases including alkaline serine protease (Moutaouakil et al. 1993; Monod et al. 1993), aspartic protease (Pep) (Reichard et al. 1994) and metalloproteases (MEPs) (Monod et al. 1993 ;Shende 2018) that help in the initial colonisation of host lung tissue. It is believed that these degradative enzymes are involved in the breakdown of ECM barrier of the host (Latgé 1999). Studies done on human lung carcinoma cell line (A549) suggest that *A. fumigatus* conidia bind and penetrate the cell surface with an efficiency of 30% (Wasylnka & Moore 2002).

Several research groups around the world have attempted to purify the conidial adhesin proteins and glycan components associated with them. However, only a handful of conidial adhesins have been identified and characterised to date in *A. fumigatus*.

Adhesion capability of *A. fumigatus* conidia with purified human ECM proteins and its associated glycan components have been demonstrated by certain studies. Annaix *et al*., have elucidated the interacting sites on human fibrinogen and *Afu* conidia, involved in the adhesion phenomenon. The D-domain of fibrinogen binds to conidial component of *A. fumigatus* in a specific and saturable manner. Proteolytic pre-treatment of conidia abolished this binding revealing the protein nature of fibrinogen interacting molecules on their surface (Annaix et al. 1992). Rod A is a GPI- anchored extracellular hydrobhobin protein present on the surface of *Afu* conidia. Presence of this extremely hydrophobic protein on conidial surface masks their recognition from host immune cells by escaping from crucial dectin-1 and dectin-2 receptors, (Blango et al. 2019), thereby RodA protein is considered conducive to the virulence. *A. fumigatus* conidial mutants (∆RodA) display decreased capability of adherence to collagen and BSA, but showed affinity for pneumocytes, fibrinogen or laminin (Thau et al. 1994; Sheppard 2011).

Besides RodA, in *A. fumigatus* at least six morehydrophobins are present namely, RodB, RodC, RodD, RodE, RodF and RodG. Out of these, only RodA and RodB are expressed significantly during sporulation and biofilm formation, respectively. However, RodA has been identified as the sole contributor to the virulence of *A. fumigatus* by imparting the characteristics of hydrophobicity, structural rigidity, immune-inertia and possibly drug-resistance as well (Valsecchi et al. 2017).

Another study carried out by *Peñalver et al*. demonstrated that *A. fumigatus* conidia can effectively adhere to the purified human fibronectin, whereas the binding of fibronectin with its mycelia was not significant. Trypsin treatment of conidia removes the adherence factors off the conidial surface, leading to reduction in binding efficacies. This suggests the proteinaceous nature of these factors. Two polypeptides of 23 kDa and 30 kDa size, purified from conidial surface extracts show interaction with fibronectin (Peñalver et al. 1996).

Affinity purification approach has also been used to identify adhesins with affinity for fibronectin and laminin (Gil et al. 1996; Tronchin et al. 1997). A 37 kDa protein identified through this approach was later characterized as *Aspergillus* allergen Aspf2 with affinity for laminin (Banerjee et al. 1998).

Interestingly, certain adhesin predictor software programms, such as, 'SPAAN', 'FungalRV' and 'FAApred' etc. have also markedly enhanced the identification, as well as the wetlab characterisation of a putative fungal adhesins and their possible role in the infection. SPAAN is based on artifical neural networks that process the adhesin probability (*P*_ad_) of a protein by analysing the amino acid, dipeptide, multiplets frequencies as well as charge and hydrobhobic composition of the proteins (Sachdeva et al. 2005). Fungal RV predicts human pathogenic fungal adhesins and adhesin like proteins based on multifaceted parameters of protein composition similar to SPAAN by employing the Hidden Markov model (HMM) matrix. (Chaudhuri et al. 2011). Another adhesin prediction tool FAApred uses the Support Vector Machine (SVM) based approach for prediction with 86.29% of accuracy (Ramana and Gupta 2010). Nath also developed an adhesin prediction model with 94.9% of accuracy by evaluating various protein sequence based features (Nath 2019).

Upadhyay *et al*. used bacterial adhesin predictor software SPAAN for identifying putative adhesins of *A. fumigatus*. From the putative adhesins identified using SPAAN, AfCalAp, an Extracellular thaumatin domain protein, was selected for *in vitro* characterisation (Upadhyay et al. 2009). The ORF of *AfCalA* was cloned and recombinantly expressed in competent *E. coli* cells. Further *in vitro* binding assays suggested that the recombinant AfCalAp has affinity for laminin, fibrinogen and murine lung cells. Furthermore, anti-AfCalAp antibodies reduced the binding affinity of AfCalAp to laminin in a dose dependent manner. Upon investigation of antigenicity and immunoreactivity of AfCalAp, ABPA patients have been found to have elevated levels of antibodies against AfCalAp (Upadhyay et al. 2009). Adhesin nature of 'AfCalA' was further studied by Liu *et al*. in 2016. By employing the gene-knock down approach, they observed that the ∆AfCalA mutants and wild type conidia adhered equally efficiently with laminin and A549 cell-line, and concluded that AfCalA is dispensable for adherence of *A. fumigatus*. However, the mutant conidia were less efficient in invading host tissues than the wild type conidia (Liu et al. 2016). Furthermore, antibodies against AfCalA improved the survival of mice model of IPA. ΔAfcalA mutant showed significantly attenuated virulence in immunosuppressed mice, as the model infected with mutant stain showed improved survival, reduced fungal burden in lung, and decreased lung tissue invasion, suggesting that AfCalA mediates pulmonary invasion, and plays an important role in the progression of invasive pulmonary aspergillosis (Liu et al. 2016).

Another predicted adhesin Mp1 (Galactomannoprotein) in *A. fumigatus* has been characterised *in vitro* by employing binding assays with purified ECM proteins. The results showed significant binding of recombinant Mp1 adhesin to laminin and fibrinogen. ABPA patients also showed high levels of IgG and IgE antibodies against Mp1 (Upadhyay et al. 2008).

Hydrofluoricacid (HF) extractable *CspA (*cell surface protein A), a GPI-anchored cell wall protein (CWP) with molecular weight of approximately 75 kDa was studied by Levdansky*et al*. (2010). *CspA* has been found to interact with two other cell-wall associated proteins namely, *Ecm33* and *Gel2. ΔcspA* mutant has been found to exhibit reduced conidial adherence to the substratum. A combined knock out strain with *ΔcspA, Δecm33* and *Δgel2* significantly reduced the conidial adherence to ECM proteins suggesting its possible role during infection. Overexpression of CspA is associated with the resistance of conidia from phagocytic cell mediated destruction (Levdansky et al. 2010).

AFUA_5G09330 is a 12–29 kDa monomeric cytosolic protein belonging to a large family of fungal proteins and identified as homolog of the CipC-like protein of *A. nidulans*. Morpho-differentiation of hyphae from conidia is a hallmark of invasive aspergillosis caused by *A. fumigatus*, AFUA_5G09330 gene is composed of two introns and has been identified as differentially expressed during hyphal stage. CipC like protein homologs have been found to exist most frequently in filamentous forms of fungi than yeasts. Seventeen proteins of the CipC family have been found in the genomes of *A. fumigatus, A. clavatus, A. flavus, A. nidulans, A. niger, A. oryzae* and *A. terreus*. AFUA_5G09330 belongs to a family of proteins that is exclusively found in fungi (Bauer et al. 2010).

UDP-galacturonate 4 epimerase3 (Uge3), a key enzyme in the pathway of GAG biosynthesis is thought to be necessary for adhesion of *A. fumigatus* hyphae to host, as GAGs are essential for adhesion. Mice infected with Δuge3 mutant strain of *A. fumigatus* lacking N-acetyl galactosamine in its cell wall showed reduced fungal lung burden and increased survival compared to those infected with wild type *A. fumigatus* strain. The attenuation of virulence and reduced fungal load is considered to be due to the reduced adherence and colony formation by this mutant strain inside the mice lungs (Gravelat et al. 2013).

### 3.2. Candida albicans

*Candida sp*. like many other pathogenic fungi isable to densely adhere to various surfaces, including the skin, endothelial tissues and epithelial mucosa of the hosts. Additionally, *Candida sp*. also adheres to inert surfaces of an object, such as denture prostheses, urinary catheters intravascular and prosthetic heart valves (Busscher et al. 2010; Pwj et al. 2013).

*C. albicans and S. cerevisiae* possess two classes of covalently linked cCWPs: the dominant class include GPI-dependent CWPs linked to beta- glucan component in the cell wall, second less abundant class is Pir-CWPs (proteins with internal repeats) also linked to beta-glucans. Extracellular cell wall adhesins Als1p and Als3p are GPI-dependent CWPs that adhere to the beta- glucans via GPI-anchor (de Groot et al. 2004).

Hwp1, ALS and EPA family of adhesins are well characterised adhesin proteins of *C. albicans*. The ALS (agglutinin-like sequence) gene family of adhesin encodes eight cell-surface glycoproteins (Als1p, Als2p, Als3p, Als4p, Als5p, Als6p, Als8p and Als9p). Als proteins consists of three domains: an extracellular N-terminal domain with adhesive function, central domain and a C-terminal domain with abundant N- and O-linked glycosylation (Kapteyn et al. 2000; Zhao et al. 2004).

Structurally related adhesins Hwp1, Als1p and Ala1p/Als5p in *C. albicans* and Epa1p in *C. glabrata* have been studied. All of these proteins belong to a class of glycosylphosphatidylinositol-dependent cell wall proteins (GPI-CWP) and have a characteristic N-terminal GPI-anchor facilitating the anchorage of Glycan residues of the cell wall (M JS et al. 2005).

Among these proteins, Als1p (Buurman et al. 1998) and Ala1p (Gaur et al. 1999), also called Als5p (Fu et al. 1998) encoded by members of a large family of ALS genes (agglutinin-like sequence) (Hoyer 2001), have been investigated to have binding affinity for ECM proteins and components of endothelial and epithelial cells (Sundstrom 1999). Δals3 mutant strain showed inefficient binding when tested against BEC and HUVEC cell lines. Adhesion data suggests that the loss of Als3p affects the adhesion of *C. albicans* more than the loss of Als1p (Zhao et al. 2004).

Hwp1 (hyphal wall protein1) gene is differentially expressed during germ tubes and hyphal stages, and encodes a mannoprotein Hwp1 containing exposed extracellular N-terminal domain (Staab et al. 1996). *C. albicans* strains lacking Hwpl has been found to form unstable adhesion to buccal epithelial cells of human and has a reduced ability to induce systemic candidiasis in mice (Staab et al. 1999). Human transglutaminase enzyme is thought to catalyse a strong and stable interaction between its substrate and human epithelial cells. The N-terminal domain of Hwp1 is supposed to mimic the transglutaminase substrate. Hwpl plays crucial role in proliferation of *C. albicans* in keratinised epithelium by interacting with keratinocyte transglutaminase enzyme (Staab et al. 1999). Moreover, surface expressions of Hwp1 have been studied to be more important in mucosal candidiasis (Kartasova et al. 1988; Balish et al. 2001). It is well known that *C. albicans* binds to different extracellular membrane proteins namely fibronectin, entactin, laminin and collagen, and are considered as potential target molecules when *Candida* spreads in the host body (Calderone 1993; Pendrak and Klotz 1995; Chaffin et al. 1998). *C. albicans* has many fibronectin receptors on its surface, including 105-kDa and 60kDa glycoproteins and mammalian homologs of integrins (Sa et al. 1994). At least three fibronectin domain(s) have been implicated in its adherence with *C. albicans*. 55 kDa and 105 kDa polypeptides have FN domain and show binding activity with fibronectin. 105kD apolypeptide additionally possesses RGD (Argenine, Glycin, and Aspartic acid) sequences. RGD tripeptide residues are involved in integrin-mediated adherence to host ECM. Many ECM proteins have been found to have the RGD sequences. Fungal pathogens contain cell surface proteins that can recognise these regions of their substrate and thereby initiate attachment to the host ECM factors. *In vitro* study has demonstrated the ability of peptide mimicking the RGD sequences of ECM protein fibronectin in inhibiting the adherence of *C. albicans* blastospores to endothelial cells (Hostetter 2000).

Although, in *C. albicans* several molecules with receptor characteristics have been described to be involved in the binding of fibronectin and laminin. An enzyme GAPDH, capable of binding to fibronectin and laminin has been studied and its location was demonstrated in *C. albicans* cell walls, using immune-electron microscopy. Being capable of binding to the exposed ECM proteins from the cell surface of *C. albicans*, GAPDH is supposed to play important part in attachment of fungal organism to the host cell thereby progressing infection (Gozalbo et al. 1998). Gozalbo and collaborators (Sa et al. 1994) have shown that the enzyme GAPDH has binding affinity for fibronectin and laminin and could be implicated in the adherence of fungal cells to host tissues, thereby, contributing in virulence. ALA1 gene of *C. albicans* conferring adherence properties towards ECM proteins, require presence of atleast four contiguous threonine residues for maximum compliance (Gaur et al. 2002).

In *C. albicans*, a hub protein that connects cellular pathways such as cell wall synthesis, biofilm production, drug resistance to antifungal agents (fluconazole and caspofungin) has been found to be involved and playing important roles in these phenomenon. Mutant strains of SMI1 gene have shown weaker cell wall architecture and susceptibility to antifungal agents. Strain that overexpress this protein has been found to have greater cell wall adhesion by many folds than the wild type strain. It is also involved in hyphal morphogenesis (Martin-Yken et al. 2018). In *C. albicans*, the EAP1 gene (a putative cell-wall adhesin) has been identified, and *in silico* analysis indicated that it consists a signal peptide, a GPI-anchor site and has homology with various genes of many yeast encoding CWPs. *Saccharomyces cerevisiae*, expressing heterologous EAP1 of *C. albicans* showed improved adherence to HEK293 cell line (Li and Palecek 2003).

### 3.3 . Candida glabrata

People with old age and compromised immune status are highly susceptible to another potent human opportunistic pathogen namely, *Candida glabrata* and is the main culprit of invasive candidiasis besides *C. albicans*, accounting for more than 20% of all blood stream candidiasis in the USA (Desai et al. 2011; Pfaller & Diekema 2007).

*C. glabrata* often survives the phagocyte(s)-mediated killing and support dissemination of infection. The ability of *C. glabrata* adherence to mammalian host tissues and various surfaces is governed by numerous surfaces expressed specific adhesins. Presence of various sequences (about 66) encoding GPI-modified adhesin-like wall proteins has been revealed from the *C. glabrata* genome. These proteins possess modular structure for adhesion as well as an effector domain followed by low complexity region with internal tandem repeats (Pwj et al. 2013). Genome wide analysis of *C. glabrata* revealed the largest family of adhesins encoding genes, further grouped into seven sub-families (I–VII) based on similarity of N-terminal domain of these adhesins. Lectin like Epithelial adhesins (Epa) are the largest sub-family of adhesins, belonging to sub-family I, consisting of 17 members in CBS138 strain (Kaur et al. 2005) and 23 members in BG2 strain (de Groot et al. 2008) of *C. glabrata*, are believed to act as important virulent factors in *C. glabrata* mediated pathogenesis (Desai et al. 2011; Pwj et al. 2013). EPA adhesins are composed of N-terminal binding (A) domain, a central variably glycosylated serine, threonine rich repeats (B) domain and a C-terminal GPI-anchored domain (Diderrich et al. 2015).

N-terminus A domain has been found to have variable specificity and discrimination for binding with host epithelial ligands such as, peptides (Cormack et al. 1999) or glycan components (Zupancic et al. 2008) on epithelial cells. Epa1, Epa6 and Epa7 are well characterised adhesins in *C. glabrata* acting as lectins and show differential binding affinity for α and β-glycans (Maestre-Reyna et al. 2012). Epa adhesins possess PA14 (Anthrax protective antigen) domain usually found in yeast adhesins (Desai et al. 2011; Pwj et al. 2013).

*In silico* genome mining analysis and characterisation revealed someof the other proteins having characteristic adhesin domains in *C. glabrata* (Desai et al. 2011). These adhesins include GPI-anchored Pwp7p (PA14 domain containing Wall Protein), Aed1p (Adherence to Endothelial cells 1) and Aed2p (Adherence to Endothelial cells 2) adhesins having adherence affinity to host endothelial cells. Interestingly, Pwp7p and Aed1p have large intragenic tandem repeats and an exclusive conserved repeat “SHITT” motif thought to be involved in fungal pathogenesis (Desai et al. 2011).

### 3.4. Cryptococcus neoformans

Cell flocculin 1 (Cfl1), first identified hypha-specific adhesin protein of *Cryptococcus neoformans*, mostly induced during *Cryptococcal* sexual development. Cryptococcal Cfl1 adhesin is unique among the most characterised adhesions of opportunistic fungi, as it contains the conserved C-terminal cystine rich, SIGC domain and N-terminal signal peptide that make it secretory.

*Cryptococcal* strain overexpressing the Cfl1 protein exerts increased flocculation, colonisation and crumpled colony morphology (Wang et al. 2012). Deletion mutation in *CPL1* gene affects capsule formation in *C. neoformans*, a potent virulence factor governing cryptococcal pathogenesis. *Cryptococcus* expresses, secreted as well as cell surface forms of Cfl1 protein, regulating paracrine intercellular communication. Cfl1 protein homologs have also been identified in *Cryptococcus* genome namely *DHA1, DHA2, CPL1* and *CFL105. DHA2* is expressed specifically in yeast form and *DHA1* in all cell types predominantly in basidia (Gyawali et al. 2017).

*C. neoformans* growth can effectively be inhibited by monocytes when grown in fibronectin coated substratum but growth remains unaffected when the interaction occurs between *C. neoformans* capsule and fibronectin. Rodriguez and his colleagues (Rodrigues et al. 2003) demonstrated that *C. neoformans* does not bind to this glycoprotein of the ECM and binding to soluble FN is also very poor compared to other fungal models. Both conidia and *S. schenckii* yeast cells were similarly attached to proteolytic fragments of FN (Lima et al. 2001). Main virulence factor involved in Cryptococcal infection is hydrated polysaccharide capsule composed of glucuronoxylomannan (GXM) and galactosylomannan (GalXM) and mannoproteins (MP) (Kumar et al. 2011). Mannoproteins (MP) accounts for only <1% of total capsular weight (Rodrigues and Nimrichter 2012; Teixeira et al. 2014). Four characterised mannoproteins (MP) – 84, 88, 98 and 115 kDa have been observed to be involved in eliciting immune response inside the host, making them subject of potent vaccine candidature against cryptococcosis (Teixeira et al. 2014).

Mannoproteins have been investigatedto be localised mainly in the inner capsule layer, in proximity to the cell wall, but not connected with GXM or GXMGal (Vartivarian et al. 1989; Jesus et al. 2010). In order to cause infection *C. neoformans* interacts with various host tissues, namely the endothelial cells, lung epithelia and the blood brain barrier (Goldman et al. 1994). Strains lacking GXM exploit other capsule components as adhesins, to adhere with host cells because mannoproteins in the inner capsule surface become exposed (Teixeira et al. 2014).

### 3.5 Other fungal pathogens

Dimorphic fungi are the main etiological agents of endemic mycoses that grow at 25°C in the form of filamentous mould, and at 37°C in the form of yeast.It has been found that SOWgp (spherule outer wall glycoprotein) adhesin of recombinant *Coccidioides immitis* is able to adhere to ECM proteins of mammals. But, after deletion of *SOWgp*, partial loss in binding of *C. immitis* spherules to ECM proteins has been recorded, that eventually leads to attenuated virulence (to some extent) in mice models (Pwj et al. 2013).

The members belonging to genus *Paracoccidioides* are the main causative agents of paracoccidioidomycosis (PCM). *Paracoccidioides lutzii* and *P. brasiliensis* are the only two species belonging to *Paracoccidioides sp*. They have characteristics that allow their growth in unfavourable conditions, allowing them to attachand invade host tissues, culminating in the severe infection. Adhesins, the CWPs facilitate adhesion and are able to mediate interactions between fungi and hosts during infection (de Oliveira et al. 2015). In case of *.brasiliensis*, their ability to infect Vero cells and the phenomenon of adherence varies depending upon the strain. Recently, a glycoprotein of 30 kDa and pI 4.9 from *P. brasiliensis* has been found to be a possible adhesin capable of adhering to laminin, and not to other components of ECM, typeI or type IV collagen fibronectin, and may contribute to the virulence of *P. brasiliensis* (Andreotti et al. 2005).

A 120 kDa glycoprotein (WI-1) is the most studied adhesin in *Blastomyces dermatitidis*. Now, it is characterised as BAD1 and has been implicated inpromoting the adherence of yeast cells to ECM components of host cells (Brandhorst et al. 2003). Hung and colleagues (Hung et al. 2002) isolated the *Coccidioides immitis* (SOWg) gene, encoding a glycoprotein component of the outer wall, specifically during the infection phase. Loss of SOWgp gene and *in vitro* binding of recombinant protein (rSOWp) to ECM proteins (namely: laminin FN collagen type IV) resulted in reduced virulence as there was partial loss in the capacity of infectious spores to adhere to ECM components of mammalians. In the cell surface extracts of *Penicillium marneffei*, a 20 kDa protein was identified and later characterised as laminin and fibronectin ligand, indicating that these two ligands share the same receptor for binding (Hamilton et al. 1999). A 50 kDa adhesin from the *Fonsecaea pedrosoi* has been characterised as a receptor, binding to mannose and to N-acetyl-D-glucosamine (Limongi et al. 2001).

## 4. Adhesins in the virulence

In order to examine the possible role of adhesins in the virulence of the fungal pathogen *C. albicans*, Rosiana *et* *al*. has created a library of 144 single and double mutant of adhesins and have been studied in *C. elegans* model. Als1ΔIff4Δ, Hyr1ΔAls5Δ, Als7ΔAls5Δ double mutants have been found to be low virulence strains and showed higher percentage worm survival in liquid killing assay upon infection with these mutant stains compared with wild type *C. albicans* stain. Upon genetic interaction analysis, the double mutant strains of adhesins Als5Δ Eap1Δ, or Als5ΔHwp2Δ conferred significant low virulence as compared to single mutant strains of these adhesin genes showing the synergistic effect of these mutations on virulence (Rosiana et al. 2021). Virulence in Paracoccidioides involve increased expression of multiple adhesin gene at different phases of infection. GP43, malate synthase, glyceraldehyde-3-phosphate dehydrogenase, enolase, 14-3-3 protein and triosephosphate isomerase are few characterised adhesins involved in host–pathogen interaction (de Oliveira et al. 2015).

## 5. Expression of adhesins in the infectious fungal morphotypes

Morphological changes are required by various eukaryotic pathogens in the process of causing disease. It is now evident, that these transitions are also associated with the progression of infection by human opportunistic fungi. The stored signalling pathways regulate the morphogenetic differentiation in response to host physiological and environmental stimuli and also change the composition of the cell surface as well as the size and shape of the cells during morphogenesis. These subtle changes are required to favour pathogenic events. Most fungi grow through unicellular budding and division or filamentously through apical expansion. Various human pathogenic fungi can switch between two or more morphotypic forms of growth, such as the unicellular and filamentous forms and therefore are called dimorphic fungi. It was established in the 'classical' dimorphic pathogens, that for virulence, a correct morphotype transition is necessary. Genetic mutations or chemical blocking these transitions weaken or negate the potential of the fungal pathogen to confer disease (Wang and Lin 2012).

The repertoire of cell surface adhesins are tightly regulated during morphotype switches: certain adhesins are solely expressed in the virulent form, while others in the saprotrophic/commensal form. Adhesin, Bad1 of *Blastomyces dermatitidis*, (a human pathogenic fungus) is expressed particularly in pathogenic yeast form and this protein is responsible for controlling multiple processes during infection (Klein and Tebbets 2007). In *C. albicans*, adhesin Hwp1 is expressed in hyphae for ensuring fungal adherence to epithelial cells, as Hwp1 adhesin proteins are important for systemic spread of candidiasis (Staab et al. 1999). *Candida* hyphae express Als3, a type of adhesin, having binding affinity for various host receptors. The binding of the receptors to the adhesin, induces its own endocytosis for facilitating penetration of fungi into epithelial cells (Zhu et al. 2012). On the contrary, *C. neoformans* adhesin Cfl1 is associated with non-pathogenic filaments, and virulence is attenuated when there is induced Cfl1expression in the form of pathogenic yeast (Wang and Lin 2012).

Taken together, changes in the composition of cell surface of fungal cells are likely to underlie the correlation between virulence and morphogenic transitions. In pathogenic fungi, normally present in open environment, the filamentous form is usually not pathogenic. Nevertheless, these adhesion molecules on the fungal morphological forms help to initiate interactions between the host and pathogen, subsequently establishing infections (Wang and Lin 2012).

Fungal colonisation in host and establishment of infection and systemic circulation is a consequence of down-regulation of filament/spore specific molecules and up-regulation of yeast specific molecules. Thus, the study of these molecules unique to each morphological form could provide new insights into the nature of host–fungal interaction (Wang and Lin 2012).

## 6. Tissue tropism exhibited by adhesins

**Figure 3.**
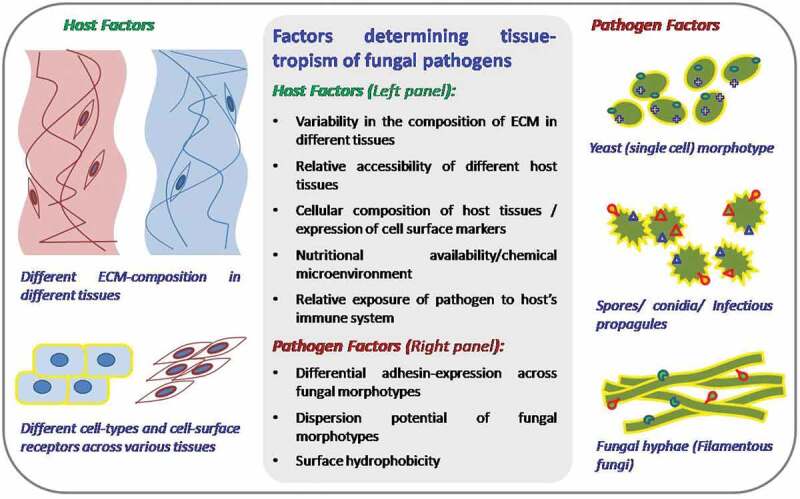
Combination of host and pathogen factors determine the tissue tropism of fungal pathogens

Relatively higher binding affinity of a pathogen or molecule towards any specific tissue is known as its tissue-tropism. Adhesins show varying degree of tissue tropism possibly depending on the molecular and cellular composition of the site of infection. Higher tropism of any adhesin towards a tissue (or its inhabitant cells) that is also a cardinal infection site for the pathogen is an important indicator of its possible role in the adhesion phenomenon. Overall tissue tropism of any fungal morphotype is determined by a combination of host and pathogen factors involved (Figure 3). Among the pathogen related factors, differential expression of adhesins in various fungal morphotypes and relative dispersion potential of these morphotypes could be important determinants.

Many fungal adhesins display morphotype-specific expression. Examples include: Hwp1 and Hwp2 adhesins of *Candida albicans* that *are expressed in* hyphae (Nobile et al. 2008; Ene and Bennett 2009; Hayek et al. 2010), Bad1, a yeast specific adhesin and important virulent factor of Blastomyces dermatitidis (Brandhorst et al. 2003) and Cell flocculin 1 (Cfl1), a hyphae-specific adhesin of *Cryptococcus* (Wang et al. 2012, 2013; Tian and Lin 2013). Similarly, AfCalAp, an adhesin of *A. fumigatus*, appears to display better expression in swollen conidia, than in resting conidia (Upadhyay et al. 2009).

On the other hand, host factors as determinants of tissue tropism may include: Different composition of ECM in various tissues (or across their developmental stages), cellular composition of host tissues and expression of cell surface markers, accessibility of different host tissues to pathogen and relative exposure of pathogen to host’s immune system.

In a tissue, cells are surrounded by ECM that constitutes the larger volume in tissue than cells it surrounds. Along with glycosaminoglycans (GAGs) linked to proteins to form the proteoglycans, the ECM is made up of many proteins, including collagen, elastin, fibronectin, lamininand fibrinogen (in damaged tissues) that are known for their adhesive and structural properties (Alberts et al. 2002). However, the compositions of ECMs vary across various tissues and their developmental stages as per the structural and functional requirement of the tissue (Alberts et al. 2002; Kular et al. 2014). As specific adhesins may have different affinities with various ECM proteins, the relative abundance of these proteins may directly influence of tissue tropism of an adhesin. Similarly, tissue tropism of an adhesin could also be influenced by cellular composition of the tissue and expression of adhesin-specific receptors on the surface of inhabitant cells. We studied tissue tropism of *A. fumigatus* protein AfCalAp by studying its binding with mice lung and spleen cell suspensions using flow-cytometry. AfCalA binds with significantly affinity with lung cell suspension, which was expected in view of *A. fumigatus* being a respiratory pathogen (Upadhyay et al. 2009). Among ECM proteins tested, AfCalA shows marked binding with laminin in comparison with fibrinogen and collagen, whereas no interaction could be seen with fibronectin and decorin in the dot-blot assay (Upadhyay et al. 2009). As revealed by another study, AfCalA also interacts with Integrin α_5_β_1_ on endothelial and epithelial cells and facilitates endocytosis mediated invasion of the fungus in host tissue (Liu et al. 2016).

In *C. albicans* hydrophilic vs hydrophobic nature of yeast cells display differential tissue tropism in mice and hydrophobic cells bound more pronouncedly to various tissues (Hazen et al. 1991). Interestingly, in this fungus, thigmotropism (sensing surface contour by hyphae) and chemotropism together are also the proposed determinants of tissue-tropism; however, more studies are required to establish these (Davies et al. 1999)

In *C. neoformans*, both acapsular (CAP67) strains and wild type (NE-241) show considerably higher tropism towards lung cancer cell-line A549, in comparison with PC3, a control prostate cancer cell line. Binding of CAP67 strain to lung cells is inhibited by a (soluble) mannoprotein-adhesin MP84, revealing its lung-tropism and role in facilitating adhesion of *C. neoformans* to lung epithelial cells (Teixeira et al. 2014).

Though adhesins of most of fungal pathogens have not been examined across the panel of various cell types of host in terms of their relative tissue tropism, numerous of them have been tested for binding with different ECM proteins (Table 1). This provides an indication of involvement of specific interactions of adhesins even within the group of ECM proteins.

## 7. Adhesins in biofilm formation

The formation of biofilms by micro-organisms, especially fungi and bacteria, has been extensively studied. Biofilms formed from pathogenic fungi have gained attention in recent years and different species like, yeast, filamentous and dimorphic fungi have been described as capable of developing in community. Biofilms are sessile fungal and microbial communities that adhere strongly to surfaces and among each other; and also, are protected by their own secreted ECM composed mainly of polysaccharides. Adherence is crucial for cells that form biofilms and the interaction between ligand and adherent is maintained by hydrophobic and electrostatic forces. Multiple adhesion molecules work in the successful creation of the biofilm.

*C. albicans* exhibits specific mechanisms of disease development that overcome the defence and allow colonisation of the mucous tissue.It has been already established that biofilm formation often relates to morphotypic switching and the degree of virulence shown by fungal cells (Baillie and Douglas 1999; Chandra et al. 2001). The expression of *C. albicans* virulence factors may also vary during different stages of infection (localised and systemic), and host immune response (Naglik et al. 2003). *C. albicans* can simply grow as biofilm on medical equipments, like urinary catheters and dentures prosthetics further increasing the chance of dissemination to other tissues of the underlying patients.

**Figure 4.**
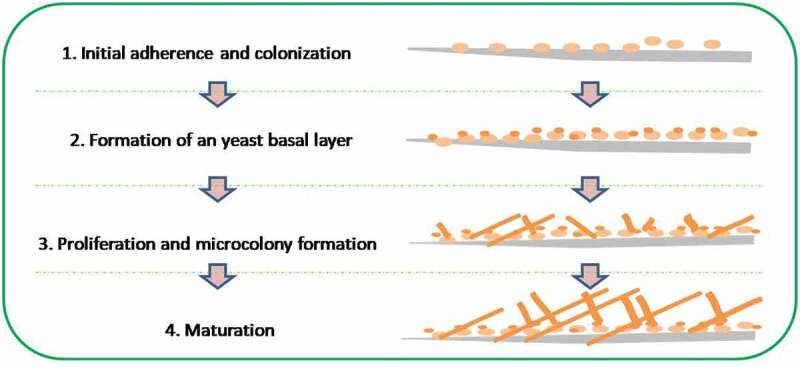
Various phases of biofilm formation by fungi

Biofilm formation involves several steps: attachment and colonisation of yeast cells to the substrate, the subsequent cell proliferation leading to formation of scaffold layer of mycelial network, accumulation and secretion of ECM, and finally dissemination of the cells to other sites (Figure 4) (Nobile and Mitchell 2006). Adherence of *Candida* cells to mucosal surfaces and/or synthetic material is an early step before they proliferate and consequently form biofilm in the process of infection (Silva et al. 2012). The presence of specific cell-wall proteins, designated normally as adhesins, is a trigger in the modulation of the adhesion process (Silva et al. 2011).

Fungal cells in maturation phase of biofilm can better withstand the antifungal therapies and host immunological factors compared within dividual fungal cells (Fanning and Mitchell 2012). The propertyof *C. albicans* biofilm formation is associated with regulatory transcription genes (JS and Mitchell 2011). These genes include BCR1, TEC1 and EFG1. The family of ALS adhesions (agglutinin-like sequence), including eight members (ALS1-ALS9), are expressed by ALS genes that encode cell surface GPI-anchored glycoproteins. In this group, ALS3 is the most prominent contributor in the biofilms formation and is usually upregulated during the infection of the oral mucosal epithelium (Murciano et al. 2012; Zordan & Cormack 2012). Some other proteins that contribute to this phenomenon are HWP1, EAP1, SSA1 lipases (LIP), phospholipases (PLBs) and proteases (SAP) (Wächtler et al. 2011;Barros et al. 2016). These proteins also promote colonisation and thereby infection through degrading the host cell barriers (Barros et al. 2016).

The Hyphal wall protein (Hwp1) is a mannoprotein of the fungal cell wall that promotes *Candida* cell attachment to the host surface (Henriques et al. 2006). It was also the first cell surface protein described as involved in the biofilm formation of *C. albicans in-vivo*. The PGA10 cell surface protein and the secreted Pbr1 protein are also described as important for the full adherence of *C. albicans* biofilms (Sahni et al. 2009). Pga10, also known as RBT51, is a member of CFEM proteins (common in many extracellular fungal membranes) and plays a role in adherence in *C. albicans* (Pérez et al. 2006). *Pérez et* *al.* have shown that Δpga10 mutants form relatively weaker biofilms leading to early detachment from the substrate, compared to the wild type strains. Furthermore, *Sahni et al*. showed that the adherence in *C. albicans* to blood cells was lower when the PBR1 gene was eliminated (Sahni et al. 2009).

Upon screening of 531 genes involved in biofilm formation from a library of conditional overexpression strains of *C. albicans*, 16 genes has been found to increase strain dominance in the multi-stain biofilm. Nine genes (*Ihd1/Pga36, Phr2, Pga15, Pga19, Pga22, Pga32, Pga37, Pga42 and Pga59*) out of 16 have been found to encode GPI- modified proteins and 8 have been found to be pathogen specific. These genes when overexpressed in the strains modulated the strain abundance and adhereance in multi-strain biofilm (Cabral et al. 2014).

Adhesion in *C. glabrata* is similar to *C. albicans*, and is achieved by epithelial adhesins (Epa) that is structurally quite similar to the Als proteins (Silva et al. 2011). Depending on the strain, the family of EPA genes consists of 17–23 genes; though, adhesins, EPA6, EPA1 and EPA7, are the very significant adhesins (Castaño et al. 2005). Deletion in the EPA1 gene reduces *in vitro* adherence of fungal cells to host epithelial cells (Cormack et al. 1999), and lactose can inhibit the adherence of this adhesin (Sundstrom 2002). Awp (Awp1-7) family of adhesins have been identified and deduced to be involved in the initial stages of biofilm development (de Groot et al. 2008). The gene expression profiling revealed that the expression of Awp adhesins are significantly higher in biofilms. *C. glabrata*, a hazardous human pathogenic fungus is capable of adhering to mammalian as well asother substrata (Cormack et al. 1999). During screening for the factors required for biofilm formation, it was identified that CgSIR4, CgRIF1 genes and protein kinase genes CgEPA6 and CgYAK1 encode for adhesins (Iraqui et al. 2005). EPA family of gene encoding for CWPs consist CgEpa6 anchored to glycosylphosphatidylinositol (Castaño et al. 2005; Domergue et al. 2005; Iraqui et al. 2005; Kaur et al. 2005). Similar to the cerebellum genes of *S. cerevisiae* FLO, sub-telomeric groups encoding the EPA genes and are underlying subject of transcriptional silencing (Castaño et al. 2005).

Glycan components of *A. fumigatus* cell wall is made up of branched and unbranched/linear β- and α-glycans, GAGs, galactomannons, chitosans, chitins and forms the structural scaffold for the wall. β- and α-glycans have shown potent role in hyphal aggregation during the formation of biofilm under *in-vitro* and *in-vivo* studies (Müller et al. 2011). α- and β-glycans synthase genes have also been found to be upregulated during biofilm formation by *A. fumigatus* (Gibbons et al. 2012). *A. fumigatus* affected patients with predisposed clinical conditions like cystic fibrosis and chronic obstructive pulmonary disease are susceptible and provides natural niches for *A. fumigatus* colonisation and biofilm formation (Ramage et al. 2011). Like other mycoses, formation of biofilm by *A. fumigatus* on epithelial cells also occurs through early, intermediate and maturation phases (Kaur and Singh 2014).

## 8. Factors regulating expression of adhesins

Studying the expression and regulation of adhesins in its various morphotypes may be important for targeting the adherence phenomenon as an anti-fungal strategy. It has been observed that adhesin-expression is a tightly regulated process controlled by different signalling pathways and in many cases the expression of adhesins is modulated in response to different environmental stimuli (such as, carbon/nitrogen starvation, changes in pH, presence/absence of ethanol in the medium, presence of suitable host-environment) and developmental stages of fungus (Guo et al. 2000; Verstrepen et al. 2003; Sampermans et al. 2005; Verstrepen and Klis 2006; Brückner and Mösch 2012; Mayer et al. 2013; Gyawali et al. 2017). These cases can be categorised as under:

### 8.1. Regulation of adhesin expression under environmental stimuli

FLO proteins are important adhesins of *Saccharomyces* required for cell-cell and cell-substrate adherence that are induced during nitrogen or carbon source starvation (Verstrepen & Klis 2006; Guo et al. 2000; Reynolds and Fink 2001). Indole acetic acid is also a stimulant for the expression of *FLO11* that is considered important for facilitation of yeast infection in wounded sites of plants (Prusty et al. 2004). In *Cryptococcus* the expression of *Dha1* adhesin is dependent on copper availability in the growth medium. It is induced when copper is limited and suppressed when copper is excessive (Gyawali et al. 2017).

In animal pathogens, adhesin expression is also induced in response to the cells perceiving an opportunistic environment for infection, enabling cells to adhere and colonise the host tissues (Verstrepen et al. 2004; Domergue et al. 2005; Verstrepen and Klis 2006). Interestingly, limitation of Nicotinic acid is used as a signal for induction of otherwise silent *Candida glabrata* adhesin genes – *EPA1, EPA6* and *EPA7*, which mediate the adherence of this fungal pathogen to uroepithelial cells in the murine model of urinary tract infection (Domergue et al. 2005).

In *C. neoformans*, extracellular secretion of *Cfl1* adhesin is used in paracrine communication as a signal for colonisation. It has been observed that, *Cfl1* is highly induced during mating colony differentiation, and some amount of this protein is released extracellularly to function as a paracrine signalling molecule. The soluble *Cfl1* protein gets enriched in the ECM and acts as an auto-induction signal to stimulate inhabitation of neighbouring cells in that region by using the filamentation signalling pathway (Wang et al. 2013).

### 8.2. Morphotype dependent expression of adhesins

Various adhesins from different fungal-pathogens show morphotype-dependent expression pattern. Hwp1 and Hwp2 proteins in *Candida albicans*, known to be crucial for adherence to host-cells and formation of biofilm, are mainly translated in hyphae; however, they also showed higher expression in opaque cells during mating (Nobile et al. 2008; Ene and Bennett 2009; Hayek et al. 2010; Gyawali et al. 2017). Als3 cell wall adhesin is specifically expressed in hyphal morphotype and plays crucial role in adherence to host epithelial cells (Gong et al. 2020). In *Cryptococcus,* Cfl1 adhesin is known to regulate the morphology, biofilm-formation and intercellular communication of the fungus. DHA1, DHA2, CPL1 and CFL105 are four additional homologs of this protein, present in *Cryptococcus*. Among these homologs, expression of Cfl1 and Dha2 is specific to hyphae and yeast respectively, whereas, Dha1 adhesin, though expressed in most cell-types, is mainly enriched at basidia. In *Blastomyces dermatitidis*, the expression of Bad1-adhesin is specific to yeast form (Brandhorst et al. 2003; Gyawali et al. 2017). Understanding the developmental stage-specificity of these adhesins in relation to predominant fungal morphotypes during different phases of infection may be important for targeting the fungal adherence by targeting stage-specific (dominant) adhesins.

### 8.3. Signalling involved in the regulation of adhesin expression

Environmental factors are known modulators of adhesin expression. These stimuli are sensed by receptors present on the surface of fungi that in response initiate signalling cascade(s) down to transcription factor(s) directly controlling the expression of individual adhesin or group of adhesins. The cAMP/PKA pathway in many fungi is important as they link development, morphotype-switching and pathogenesis. In *A. fumigatus*, deleting gpaB or acyA (cAMP/PKA pathway), results in reduced growth and conidiation (Liebmann et al. 2003). Similarly, few more studies also implicate other components of this pathway, such as PkaR (regulatory protein) and PkaC1 (catalytic protein) as crucial for proper growth, germination and precise conidiation (Lin et al. 2015). Signalling pathways involved in adhesin expression are not well defined in literatures. However, a handful of transcriptional regulators are known in the human pathogenic fungi that modulates the adhesin expressions in experimental conditions. In general, signalling cascade involving cAMP, MapK, protein kinases are associated with host–pathogen interaction and adhesin transcription factors regulation, which in turn are implicated in regulation of adhesin expression (M JS et al. 2005).

In *C. albicans*, a rapamycin sensitive protein kinases TORC1 is known to regulate the expression of adhesins, biofilm formation. Also TORC1 signalling is involved in the transcriptional regulation of many biochemical pathways in response to nutritional niche changes (Flanagan et al. 2017). TORC1 is regulated by the upstream GTPase enzymes Rhb1 and Gtr1. Double mutants ∆Rhb1 and ∆Gtr1 strains have shown hyperflocculation of biofilm and upregulation of genes involved in biofilm formation such as ALS and HWP1 genes (Flanagan et al. 2017). Upon activation of TOR1 with rapamycin in *C. albicans* increased hyphal transition and cell-cell adherence has been observed (Kim et al. 2019). A zinc-finger protein transcription factor Bcr1p is also required for biofilm formation in *C. albicans*. The expression analysis showed that Bcr1p activates the genes of the cell surface protein and adhesins. The expression of *bcr1* depends on the regulatory protein Tec1p for hyphal differentiation and is a component of down-stream regulatory network for hyphal differentiation affecting the expression of genes encoding cell surface proteins and adhesins. Out of the 22 genes whose expression is most severely affected in the ∆bcr1/∆bcr1 mutant, 11 specify cell surface proteins or enzymes that alter the cell wall. In particular, the expression of several genes of GPI-linked CWPs in mutant ∆bcr1/∆bcr1, for example HYR1, CEPE1, RBT5, ECM331 and adhesins HWP1 and ALS3 are found to be altered. Bcr1p is required for the expression of several genes that govern the properties of the cell surface. The analysis of gene expression showed a high expression of HWP1 and ALS, especially the family of ALS1 genes that showed increased expression and is characteristic of biofilm cells, compared to free cells (Nobile and Mitchell 2005).

HWP1 is supposed to be the first identified morphogenic target located downstream of the signalling machinery and hyphal development regulators in *C. albicans*. The expression of HWP1 is also influenced by the negative regulators: TUP1 and RBF1. Single and double locus deletions of HWP1 gene results in a moderate conditional defect in the development of hyphae. The expression of HWP1 has been found to be blocked in a ∆EGF1 mutant, as EFG1 positively regulates development of hyphae by encoding a basic helix-loop-helix-type transcription factor. Deletion in EFG1 manifested elongated rod like cells under the controlled laboratory conditions and unable to produce true hyphae. Down-regulation of RBF1 gene in ∆RBF11 mutants results in stimulated filamentous growth revealing its negatively regulatory role in spore to hyphae transition. TUP1 is considered to be involved in the repression of functional genes responsible for differentiation of filament and promoting yeast form. This was evident while studying the deletion mutants of TUP1 homolog in *C. albicans* resulted in pseudo-hyphal morphology under different conditions for growth. HWP1 works in downstream of the developmental modulators, TUP1, EFG1 and RBF1 during morphotypic advancement of *C.* *albicans* (Sharkey et al. 1999).

MedA protein identified in *A. nidulans* is regulated during development of hyphae and is characterised to be a temporal modulator of central conidiation gene expression by an unknown mechanism. *A. nidulan*, medA mutants have shown delayed phailide formation as well as lesser number of conidia (Clutterbuck 1969; Aguirre 1993; Busby et al. 1996; Gravelat et al. 2010). The mutant *A. fumigatus* MedA has been found in conditions of deterioration, in the formation of conidia and biofilms, as well as in the adhesion of pulmonary epithelial cells, in lesions and *in vitro* stimulation. ΔMedA mutant strains showed attenuated virulence in an AI model of invertebrates and mammals (Gravelat et al. 2010).

Zinc finger transcription factor, Znf2 is the master regulator of morphogenesis and virulence mediated by Cfl1 adhesin protein. Znf2 dictates cell adhesion, filamentation, and sporulation. *Cryptococcus* strain overexpressing Znf2 elicits defensive host immune response and drops the skill to cause deadly diseases in murine models. Cfl1 is upregulated during the transition from yeast to hypha and covers the surface of the hyphae and contagious spores generated by the hyphae. The overexpression of Cfl1 in yeast cells significantly alters the degree of virulence caused by *Cryptococcus*. Therefore, Cfl1, which naturally covers the surface of the infectious propagules of *Cryptococcus*, is implicated in the host–pathogen interaction (Wang et al. 2012).

The Cryptococci yeast culture age and the growth temperature influence the cryptococcal adherence to the pulmonary epithelial cells without alteration in yeast capsule size (Merkel and Scofield 1997). Exposed Cell surface adhesins of *C. neoformans* capable of binding lung and glial cells are fully expressed during the late stationary phase when the yeasts are grown at 37°C (Merkel and Scofield 1993).

## 9. Adhesins as potential targets for prevention and therapy

Immunisation with the recombinant N-terminal domain of Als1p adhesin (rAls1p-N) protected BALB/cas well as outbred mice not only from multiple virulent strains of *Candida albicans* but from those of non- *albicans* sp. as well (Ibrahim et al. 2005, 2006; Spellberg et al. 2005). Presence of Als family genes in other *Candida sp*. have been considered responsible for this 'across the species' immunity against *Candida* (Ibrahim et al. 2006).

A study demonstrating the effects of *A. fumigatus* on the actin cytoskeleton of pulmonary A549 pneumocytes showed that out of the three main classes of cytoskeleton fibres: microtubules, microfilaments and intermediate filaments, only actin filament undergo significant structural modification in response to infections, including actin aggregate formation, thrashing actin stress fibre, cell blebbing and impaired focal adhesion sites. These alterations could be specifically blocked in *A. fumigatus* wild type strains by adding 'antipain', a cysteine/serine protease inhibitor, while, the *A. fumigatus strain* deficient in serine alkaline protease showed insensitivity towards antipain. Approximately fifty percent reduction in fungal-induced A549 cell detachment to plate was observed in the presence of 'Antipain'. Now it is known that *A. fumigatus* breaks the barrier of alveolar epithelial cells through secreting proteases to act together to disorganise actin filament and devastate cell adhesion to the substrate by changing focal adhesions (Tv et al. 2004). Antibodies against another *A. fumigatus* adhesin AfCalA reduced its binding with laminin in a dose dependent manner (Upadhyay et al. 2009). AfCalA is absent in resting conidia but in the swollen conidia the protein is surface localised (Upadhyay et al. 2009). Interestingly, in the study of Liu *et al.*, AfCalA knockout mutant showed similar level of binding with A549 cells and laminin as that of wild type strain. However, pretreatment with anti-AfCalA significantly reduced the endocytosis of *A. fumigatus* by epithelial and vascular endothelial cells (Liu et al. 2016).

In order to promote their adhesion to epithelial surface, *Candida sp*. express adhesins linked covalently to the β-glucan residues on their cell wall. Adhesins such as, -like sequence (ALS) protein and the hyphal-1 wall (HWP1) protein, contribute in formation of the biofilm and facilitating cell surface adhesion (Nobile et al. 2006a, 2006b). Another key regulatory zinc finger protein named BCR1 is found to play important role in formation of biofilm by stimulating the expression of genes related to other CWP that are involved in formation ofbiofilm *in vivo* and *in vitro* experiments. Mutation studies on Bcr1 target genes has shown the Als1 and Hwp1 has significant role in biofilm formation. These Bcr1 target genes can be studied in more detail to draw the better comprehension of the role of these genes in biofilm formation and their therapeutic potential for prevention of the candida infection (Nobile et al. 2006a, 2006b). A hyphae specific adhesin *Als3* has been found to be a potential target of recombinant human Serum Amylase A1 acute phase protein. Rh-SAA1 protein induces cell aggregation and death in *C. albicans. Als3* double mutant strains showed defective cell aggregation upon treatment with Rh-SAA1 protein and significant higher survival rate. However, reconstituted Als3 strain showed same degree of cell aggregation and death as wild type *C. albicans* strain. This study suggested the targetebility of Als3 by Rh-SAA1 protein can greatly reduce the infection (Gong et al. 2020).

In *Blastomyces dermatitidis*, disruption of WI-1 locus resulted in loss of adherence to lung tissue and macrophages, reduced entry of the pathogen into macrophages, as well as decreased virulence in mice model. Reconstitution of WI-1 restored the virulence of *B. dermatitidis* back to normal (Brandhorst et al. 1999).

In coping with macrophages, some pathogenic fungi have also developed the ability to resist phagocytosis, as evidenced by *C. neoformans*, which can switch to a variant of the mucosal colony producing a larger GXM capsular polysaccharide, thus reducing the phagocyte efficacy of alveolar macrophages (Fries et al. 2001). Targeting the polysaccharide capsular proteins, transcription factor Znf2 and Cfl1 adhesin protein could potentially reduce the severity of cryptococcal infections (Gyawali et al. 2017). *In vitro* adhesion of cryptococci and other yeasts with purified glycosphingolipids has already been studied. These studies showed that the Glycosphingolipids, particularly lactosylceramide, may be potential receptor targets on the surface of mammalian cells for yeast morphotypes (Jimenez-Lucho et al. 1990). Cryptococci yeast cells treated with trypsin for 30 minutes lose the capability of adherence to the lung epithelial and glial cells. Formalin treatment of yeasts does not alter cryptococcal adherence to glial and pulmonary epithelial cells indicating resistance to the treatment. By contrast, treating the yeasts with trypsinabrogated their *in vitro* adherence capacity to glial and lung cells (Merkel and Scofield 1993).

## 10. Inferences

Fungal pathogenesis is a multifactorial phenomenon, therefore, it is necessary to study the nature of fungal pathogens, their virulence factors and their interaction with host defence components to develop effective antifungal therapies. Although phenomenal progress has been made with respect to the molecular characterisation of various factors of virulence and their interaction with the host; there is a need for further investigation to find out the exact contribution of each virulence factor in the different disease states. Adhesins are important cell surface components, playing important roles in adhering to other cells, or to substrates, live or dead. They have gained interest due to their virulent nature and ability to cause IFIs. Availability of modern genomic and proteomic tools has made it possible to expedite the identification and characterization of the fungal adhesins in recent years. Few studies has imparted the significant role of adhesin mutant fungal strains having reduced adherent ability and subsequent virulence in the mice models. Various studies as mentioned above have established the capability of adhesins targetability for prevention of infection and therapy. As the adhesion phenomenon is multifactorial, the adhesin families in these opportunistic fungi express differentially and coordinate the adhesion phenomenon to host tissues or other surfaces. However, the clear understanding of fungal colonisation and persistence inside the host is still lacking. In future studies on characterisation of new putative adhesins and exploring their mechanism of action would provide their involvement and role the fungal pathogenesis. Importantly, there is a grave need to screen small molecule libraries to identify drug molecules that inhibit or block the interaction of adhesins with ECMs to realise their application potential as antifungal vaccines or therapies.
